# Predictors of hospital readmission for patients diagnosed with delirium: An electronic health record data analysis

**DOI:** 10.1111/acps.13523

**Published:** 2022-12-28

**Authors:** Michaela‐Elena Friedrich, Gayan Perera, Lisa Leutgeb, David Haardt, Richard Frey, Robert Stewart, Christoph Mueller

**Affiliations:** ^1^ Department of Child and Adolescent Psychiatry Klinik Hietzing Vienna Austria; ^2^ Department of Psychiatry and Psychotherapy Klinik Floridsdorf Vienna Austria; ^3^ King's College London, Institute of Psychiatry, Psychology and Neuroscience London UK; ^4^ Department of Psychiatry and Psychotherapy Medical University of Vienna Vienna Austria; ^5^ South London and Maudsley NHS Foundation Trust London UK

**Keywords:** delirium, dementia, readmission, risk factors

## Abstract

**Introduction:**

Delirium is an acute and fluctuating change in attention and cognition that increases the risk of functional decline, institutionalisation and death in hospitalised patients. After delirium, patients have a significantly higher risk of readmission to hospital. Our aim was to investigate factors associated with hospital readmission in people with delirium.

**Methods:**

We carried out an observational retrospective cohort study using linked mental health care and hospitalisation records from South London. Logistic regression models were used to predict the odds of 30‐day readmission and Cox proportional hazard models to calculate readmission risks when not restricting follow‐up time.

**Results:**

Of 2814 patients (mean age 78.9 years SD ±11.8) discharged from hospital after an episode of delirium, 823 (29.3%) were readmitted within 30 days. Depressed mood (odds ratio (OR) 1.34 (95% confidence interval (CI) 1.08–1.66)), moderate‐to‐severe physical health problems (OR 1.67 (95% CI 1.18–2.2.36)) and a history of serious circulatory disease (OR 1.29 (95% CI 1.07–1.55)) were associated with higher odds of hospital readmission, whereas a diagnosis of delirium superimposed on dementia (OR 0.67 (95% CI 0.53–0.84)) and problematic alcohol/substance (OR 0.54 (95% CI 0.33–0.89)) use were associated with lower odds. Cox proportionate hazard models showed similar results.

**Conclusion:**

Almost one‐third of patients with delirium were readmitted within a short period of time, a more detailed understanding of the underlying risk factors could help prevent readmissions. Our findings indicate that the aetiology (as alcohol‐related delirium), the recognition that delirium occurred in the context of dementia, as well as potentially modifiable factors, as depressed mood affect readmission risk, and should be assessed in clinical settings.

**Significant outcomes**

- Almost one‐third of patients with delirium were readmitted within 30 days.
- Depressed mood was associated with an increased re‐admission risk.
- Recognition that delirium occurred in the context of dementia was associated with a lower readmission risk.

**Limitations**

- As we only included patients referred to mental health liason services, those patients might have a more severe form of delirium.
- Limited information on the nature and etiology of the delirium was available.
- Diagnoses of delirium were made according to ICD‐10 criteria, and it was not possible to ascertain whether structured screening or assessment tools for delirium were used.

## 1 INTRODUCTION

Delirium is an acute and fluctuating change in attention and cognition that increases the risk for functional decline, institutionalisation and death in hospitalised patients.^1^ The causes of delirium are multifactorial and include patient vulnerability factors (dementia or cognitive impairment, ageing, medical comorbidity, malnutrition, history of alcohol abuse/addiction as well as benzodiazepine or opioid prescription and addiction) and potentially modifiable precipitating factors such as infections, dehydration, electrolyte abnormalities, polypharmacy, seizures and surgery.^2^, ^3^, ^4^ Delirium is a common complication, especially after cardiac surgical procedures and is associated with increased morbidity and mortality.^5^ Delirium is a significant predictor and oftentimes misdiagnosed independent risk factor for 30‐day hospital readmission, emergency department visits, and discharge to a location other than home.^6^, ^7^ Postoperative delirium specifically has been found to be associated with higher risk of readmission (8; 9; 10), reoperation^8^ and increased length of hospital stay.^9^ Patient characteristics may also determine readmission rates following delirium, including primary diagnosis, sociodemographic factors, social connectedness, disabilities, difficulties with activities of daily living, cognition and functional status.^10^, ^11^ Functional network disintegration is common in patients with dementia and also yields a higher risk of developing delirium.^12^ Therefore, dementia and delirium seem to share a pathway, leaving this patient group more vulnerable for delirium than their non demented counterparts.

The aim of this study was to identify factors associated with a higher risk of hospital readmission following an episode of delirium. We hypothesised that patients with delirium superimposed on dementia and frailer patients, as reflected in a higher age, more co‐morbid physical illness and polypharmacy, would be at a higher risk of hospital readmission.

## 2 MATERIAL AND METHODS

### 2.1 Data source

We assembled an observational retrospective cohort study using anonymised data from South London and Maudsley (SLaM) NHS Foundation Trust electronic health records. South London and Maudsley NHS Foundation Trust is one of Europe's largest providers of secondary mental healthcare, serving a population of approximately 1.36 million residents in four London boroughs (Lambeth, Southwark, Lewisham and Croydon) as a near‐monopoly source of comprehensive secondary mental healthcare including inpatient, community, general hospital liaison, and forensic services.^13^, ^14^

From 2006 onwards, electronic clinical records have been used comprehensively across all SLaM services, and in 2008, the Clinical Record Interactive Search (CRIS) system supported by SLaM's NIHR Biomedical Research Centre for Mental Health was developed to enable researchers to retrieve anonymised electronic health records. CRIS contains full but anonymised information from over 500,000 mental health care users. This data resource has been previously described in detail.^14^, ^15^, ^16^ CRIS is approved as a dataset for secondary analysis (Oxfordshire Research Ethics Committee C, reference 18/SC/0372).

Data were extracted both from structured fields routinely completed in the source record and from clinical documents. Bespoke natural language processing algorithms were used to identify relevant information from free‐text records using General Architecture for Text Engineering (GATE) software.^17^ Further, CRIS is linked to national data on hospitalisations (Hospital Episode Statistics (HES)) (NHS Digital) enabling the cohort ascertainment described below.

### 2.2 Sample

We ascertained patients who received a diagnosis of delirium with or without dementia, code F05.0 or F05.1 according to ICD‐10 criteria,^18^ by general hospital liaison psychiatry services between 1 January 2008 and 28 February 2018. If patients had several delirium episodes in this window, only the first episode was considered. From linked HES data the discharge date for the hospitalisation during which the delirium episode occurred was extracted, which served as the index date for the outcome analyses.

### 2.3 Outcome criteria

#### 2.3.1 Readmission

From linked HES data we ascertained the date of any readmission to hospital at least 1 day after discharge from the hospitalisation with a delirium episode. Thus, transfers to other hospitals were excluded. Patients who died outside hospital within 30 days after discharge were also excluded and we considered two outcomes: first, we explore whether or not patients were readmitted within 30 days of discharge from the hospitalisation (i.e., as a binary outcome); second, the full cohort was followed in a survival analysis until a rehospitalisation, death, or a censoring date of 31 March 2018.

#### 2.3.2 Predictors of readmission

During the index‐admission, we ascertained socio‐demographic factors (age at discharge, gender, marital status) dichotomised to cohabiting and non‐cohabiting, ethnicity dichotomised into white and non‐white, and a neighbourhood‐level index of multiple deprivation^19^ from structured fields and supported by natural language processing applications. We extracted mental health symptoms and functioning from the Health of the Nation Outcome Scales (HoNOS),^20^ an instrument, routinely used in UK mental health services, and we dichotomised subscales to “no problem or minor” (scores 0–1) and “mild to severe problems” (scores 2–4).

For example, for depressed mood we used the HoNOS “Problems with depressive symptoms” subscale, whereby patients with “nil mood disturbance” or “gloomy or minor or transient changes in mood” (score 0 and 1) are considered not to have a depressed mood, and those with “definite depression on subjective and objective measures” (score 2) or “marked or severe depressive symptoms” (score 3–4) as having a depressed mood.^20^

To have a more fine‐grained measure of physical health, we grouped the HoNOS subscale for physical illness or disability into “no or minor” (score 0–1), “mild” (score 2), and “moderate to severe” (score 3–4) categories and further extracted from HES records whether patients had hospital admissions with a circulatory disease (ICD‐10 diagnoses: I20‐25, I50, I60‐69) in the 2 years before the index date. We further determined the presence of dementia, either on the basis of the delirium being coded as superimposed on dementia (ICD‐10 code: F05.1) or based on a previously assigned diagnosis of dementia in CRIS. An NLP algorithm was used to extract data on medications recorded in text fields in a window from 6 months before to 6 months after the index date as a measure of prevalent prescribing. We estimated polypharmacy on the basis of five or more different medications received during that period,^21^, ^22^ and also ascertained use of an antipsychotic, an antidepressant, or a hypnotic.

### 2.4 Statistical analyses

We used STATA 15 software (Stata Corp LP, College Station, TX, USA) for all analyses. Patients were grouped according whether they and a re‐admission within 30 days and groups were compared using t‐tests and chi^2^ tests. To identify factors associated with hospital readmission, we used two different approaches. First, we applied logistic regression models to investigate the odds of 30‐day readmission. Second, Cox proportionate hazard models were used to calculate the risk of readmission using the date of death or 31 March 2018 as censoring points. For both approaches we created two multivariable regression models: Model 1 was adjusted for age at discharge and gender. Model 2 was adjusted for 17 confounders: age, gender, ethnicity, marital/cohabiting status, deprivation score, all HoNOS scores, and hospitalisation with circulatory disease in the previous 2 years. Of the whole sample 22% had missing data on at least one of the covariates. We assumed missingness to be random and imputed missing values using chained equations to maximise statistical power^23^ creating 22 imputed datasets. Rubin's rules^24^ were applied to combine coefficients in the final analyses.

## 3 RESULTS

We ascertained 3007 patients with a delirium episode, of whom 8 were excluded who were readmitted on the same day and 185 who died within 30 days of discharge from hospital without being readmitted. The final sample consisted of 2814 patients with a mean age at discharge of 78.9 (SD ± 11.8) years. Of these 823 (29.3%) were readmitted within 30 days.

Characteristics of the full cohort and those grouped by 30‐day readmission status are presented in Table 1. No differences in sociodemographic characteristics were detected between the two groups. Amongst those readmitted within 30 days there was a higher proportion of patients with depressed mood, moderate‐to‐severe physical health problems, and a history of serious circulatory disease. Further the proportion of those receiving a diagnosis of delirium superimposed on dementia was lower in the re‐admitted group.

**TABLE 1:** Characteristics of the cohort of patients with diagnosed delirium and by 30‐day readmission status

	Full cohort (*n* = 2814)	Re‐admitted within 30 days (*n* = 823)	Not re‐admitted within 30 days (*n* = 1991)	*p*‐value^a^
Sociodemographic variables				
Age at discharge (mean, SD)	78.9 (11.8)	78.8 (12.2)	78.9 (11.7)	0.879
Female (%)	56.6%	54.3%	57.4%	0.132
Non‐white ethnicity (%)	18.5%	18.1%	18.7%	0.718
Married or cohabiting (%)	28.6%	29.9%	28.1%	0.354
Index of multiple deprivations (mean, SD)	28.0 (11.1)	28.2 (11.4)	27.9 (11.0)	0.467
Mental health problems according to HoNOS^b^				
Agitated behaviour (%)	41.7%	39.7%	42.5%	0.190
Non‐accidental self‐injury (%)	3.4%	3.6%	3.2%	0.608
Alcohol and/or substance use (%)	4.3%	2.8%	5.0%	0.019
Cognitive problems (%)	78.6%	76.7%	79.3%	0.153
Hallucinations or delusions (%)	32.8%	31.7%	33.2%	0.493
Depressed mood (%)	19.4%	**22.8%**	**18.0%**	**0.007**
Functional problems according to HoNOS^b^				
Relationship problems (%)	25.0%	25.5%	24.7%	0.683
ADL problems (%)	79.0%	79.9%	78.7%	0.504
Living conditions (%)	16.6%	15.5%	17.0%	0.377
Problems with daytime activities (%)	38.5%	38.5%	38.6%	0.962
Physical health and co‐morbidity				
Physical illness scale (HoNOS)^b^				**0.001**
No to minor problem (%)	9.1%	6.7%	10.1%	
Mild problem (%)	26.6%	23.9%	27.7%	
Moderate to severe problem (%)	64.3%	69.4%	62.2%	
History of serious circulatory disease (%)^c^	70.1%	**73.8%**	**68.6%**	**0.006**
Dementia diagnosis				
Diagnosis of delirium superimposed on dementia (%)	17.9%	**14.1%**	**19.5%**	**0.001**
Dementia in record (%)	38.6%	36.7%	39.4%	0.176
Pharmacotherapy^d^				
Polypharmacy (≥5 medications) (%)	22.9%	21.9%	23.3%	0.410
Antipsychotic (%)	13.9%	12.3%	14.6%	0.110
Antidepressant (%)	14.4%	14.1%	14.5%	0.799
Hypnotic (%)	11.9%	10.8%	12.4%	0.236

*Note*: Bold: *p* < 0.05.

Abbreviation: HoNOS, Health of the Nation Outcome Scale.

^a^ t‐test or chi^2^ test.

^b^ Closest to discharge date.

^c^ In the 2 years before discharge date.

^d^ In a one‐tear window around discharge date.

### 3.1 Factors associated with 30‐day re‐admission

Odds ratios and confidence intervals in logistic regression analyses of readmission within 30 days are presented in Table 2. No associations were found between sociodemographic variables or functional difficulties and 30‐day readmission. Both in the age and gender adjusted model 1 and in model 2 adjusting for 17 potential confounders, depressed mood, moderate‐to‐severe physical health problems and a history of serious circulatory disease were associated with higher odds of hospital re‐admission. A diagnosis of delirium superimposed on dementia and problematic alcohol/substance use were associated with lower odds of 30‐day readmission in both models. Receipt of an acetylcholinesterase inhibitor was associated with lower readmission odds in the age and gender adjusted model, but this was no longer significant after further confounder adjustment (OR: 0.71; 95% CI: 0.48–1.05; *p* = 0.087). No association was found with polypharmacy in either regression model.

**TABLE 2:** Factors associated with 30‐day readmission in logistic regression models (OR, 95% CI)

	Model 1	Model 2
Sociodemographic variables		
Age at discharge (1‐year increase)	1.00 (0.99–1.01)	1.00 (0.99–1.01)
Female gender	0.88 (0.75–1.04)	0.88 (0.74–1.05)
Non‐white ethnicity	0.97 (0.78–1.20)	0.99 (0.80–1.24)
Married or cohabiting	1.09 (0.90–1.32)	1.05 (0.86–1.27)
Index of multiple deprivations (per 10 point increase)	1.03 (0.95–1.11)	1.03 (0.96–1.11)
Mental health problems according to HoNOS^a^		
Agitated behaviour	0.89 (0.74–1.05)	0.92 (0.76–1.12)
Non‐accidental self‐injury	1.11 (0.68–1.81)	1.07 (0.65–1.79)
Alcohol and/or substance use	**0.54 (0.33–0.89)**	**0.57 (0.34–0.94)**
Cognitive problems	0.85 (0.69–1.05)	0.84 (0.68–1.05)
Hallucinations or delusions	0.94 (0.78–1.14)	0.95 (0.77–1.16)
Depressed mood	**1.34 (1.08–1.66)**	**1.26 (1.00–1.58)**
Functional problems according to HoNOS^a^		
Relationship problems	1.04 (0.85–1.27)	1.12 (0.90–1.39)
ADL problems	1.06 (0.85–1.33)	0.95 (0.74–1.22)
Living conditions	0.88 (0.69–1.12)	0.91 (0.71–1.17)
Problems with daytime activities	1.00 (0.83–1.20)	0.94 (0.77–1.15)
Physical health and co‐morbidity		
Physical illness scale (HoNOS)^a^		
Mild problem versus no/minor problem	1.39 (0.90–1.87)	1.29 (0.88–1.87)
Moderate to severe problem versus no/minor problem	**1.67 (1.18–2.36)**	**1.65 (1.15–2.36)**
History of serious circulatory disease^b^	**1.29 (1.07–1.55)**	**1.29 (1.07–1.55)**
Dementia diagnosis		
Diagnosis of delirium superimposed on dementia	**0.67 (0.53–0.84)**	**0.71 (0.55–0.90)**
Dementia in record	0.89 (0.74–1.05)	0.95 (0.79–1.14)
Pharmacotherapy^c^		
Polypharmacy (≥5 medications)	0.92 (0.75–1.12)	0.94 (0.77–1.16)
Antipsychotic	0.82 (0.64–1.05)	0.89 (0.69–1.14)
Antidepressant	0.98 (0.77–1.23)	0.98 (0.77–1.24)
Hypnotic	0.85 (0.66–1.11)	0.95 (0.73–1.24)

*Note*: Bold: *p* < 0.05. Model 1: adjusted for age and gender. Model 2: adjusted for age at discharge and gender and model 2 adjusted for age, gender, ethnicity, marital/cohabiting status, deprivation score, all HoNOS scores, and history of serious circulatory disease.

Abbreviation: HoNOS, Health of the Nation Outcome Scale.

^a^ Closest to discharge date.

^b^ In the 2 years before discharge date.

^c^ In a one‐tear window around discharge date.

### 3.2 Factors associated with readmission in Cox regression models

Over the full follow‐up period, 82.5% of the cohort (*n* = 2312) were readmitted, with a median time to first readmission of 100 days (interquartile range 22 to 404 days). In the age and gender adjusted model, higher age, higher deprivation, depressed mood, more severe physical health problems and hospitalised circulatory disease were associated with a higher risk of readmission, and female gender, problems with alcohol/substance use and problematic living conditions were associated with a significantly reduced readmission hazard (see Table 3). After further confounder adjustment in model 2, male gender, higher deprivation, depressed mood and a hospitalised circulatory disease remained statistically significant risk factors for readmission, whereby alcohol/substance was associated with a lower risk.

**TABLE 3:** Factors associated with re‐admission in Cox regression models (HR, 95% CI)

	Model 1	Model 2
Sociodemographic variables		
Age at discharge (1‐year increase)	**1.01 (1.00–1.01)**	1.00 (1.00–1.01)
Female gender	**0.83 (0.76–0.90)**	**0.83 (0.77–0.91)**
Non‐white ethnicity	1.02 (0.92–1.14)	1.02 (0.92–1.14)
Married or cohabiting	1.06 (0.96–1.16)	1.03 (0.94–1.14)
Index of multiple deprivations (per 10‐point increase)	**1.04 (1.00–1.08)**	**1.04 (1.00–1.08)**
Mental health problems according to HoNOS^a^		
Agitated behaviour	0.95 (0.87–1.04)	0.98 (0.88–1.08)
Non‐accidental self‐injury	0.84 (0.66–1.07)	0.84 (0.65–1.09)
Alcohol and/or substance use	**0.69 (0.55–0.86)**	**0.71 (0.57–0.89)**
Cognitive problems	0.91 (0.81–1.01)	**0.89 (0.79–1.00)**
Hallucinations or delusions	1.00 (0.91–1.09)	0.99 (0.90–1.09)
Depressed mood	**1.14 (1.02–1.27)**	**1.14 (1.02–1.29)**
Functional problems according to HoNOS^a^		
Relationship problems	0.94 (0.85–1.05)	1.01 (0.91–1.28)
ADL problems	1.02 (0.91–1.14)	1.02 (0.90–1.16)
Living conditions	**0.82 (0.72–0.93)**	0.86 (0.75–0.98)
Problems with daytime activities	0.93 (0.85–1.02)	0.92 (0.84–1.01)
Physical health and co‐morbidity		
Physical illness scale (HoNOS)^a^		
Mild problem versus no/minor problem	**1.19 (1.01–1.41)**	1.15 (0.97–1.36)
Moderate to severe problem versus no/minor problem	**1.21 (1.03–1.41)**	1.15 (0.97–1.35)
History of serious circulatory disease^b^	**1.42 (1.29–1.56)**	**1.39 (1.26–1.53)**
Dementia diagnosis		
Diagnosis of delirium superimposed on dementia	**0.83 (0.74–0.93)**	0.89 (0.80–1.00)
Dementia in record	0.98 (0.90–1.06)	1.01 (0.93–1.11)
Pharmacotherapy^c^		
Polypharmacy (>5 medications)	1.03 (0.93–1.13)	1.04 (0.94–1.15)
Antipsychotic	0.91 (0.81–1.03)	0.95 (0.84–1.08)
Antidepressant	1.06 (0.94–1.19)	1.05 (0.93–1.18)
Hypnotic	0.99 (0.87–1.12)	1.03 (0.91–1.17)

*Note*: Bold: *p* < 0.05. Model 1: adjusted for age and gender. Model 2: adjusted for age at discharge and gender and model 2 adjusted for age, gender, ethnicity, marital/cohabiting status, deprivation score, all HoNOS scores, and history of serious circulatory disease.

Abbreviation: HoNOS, Health of the Nation Outcome Scale.

^a^ Closest to discharge date.

^b^ In the 2 years before discharge date.

^c^ In a one‐tear window around discharge date.

A diagnosis of delirium superimposed on dementia was associated with a lower re‐admission hazard in model 1, but no longer after further confounder adjustment (Hazard ratio (HR): 0.89, 95% CI: 0.180–1.00; *p* = 0.056). Notably, in model 2 the presence of cognitive problems at discharge was associated with a lower risk of readmission (HR: 0.042; 95% CI: 0.79–1.00; *p* = 0.042). A prior recorded dementia diagnosis in the patient record was not associated with readmission in either logistic or Cox regression models. None of the medication variables examined, including polypharmacy, were significantly associated with readmission in the Cox regression models.

## 4 DISCUSSION

We examined factors associated with readmission in close to 3000 patients diagnosed with delirium by general hospital liaison psychiatry services, of whom almost one third was re‐admitted within 30 days. Besides the expected associations of physical co‐morbidity with readmission, our most consistent finding was the presence of depressed mood associated with a higher risk of readmission. Other than acetylcholinesterase inhibitors being associated with lower odds of 30‐day readmission in minimally adjusted model, pharmacotherapy did not seem to play a role. Interestingly, a documented diagnosis of delirium superimposed on dementia yielded a lower readmission risk.

While delirium itself is a well‐established risk factor for hospital readmission,^25^ only one other study has to our knowledge evaluated re‐admission rates after an episode of delirium. Elsamadicy et al.^26^ described 30 day‐ readmission rates of 12% in patients with postoperative delirium after spine surgery. Our higher rates may reflect the wider inclusion of all forms of delirium and a sample of patients referred to hospital liaison services.

Within our sample, depressed mood was significantly associated with 30‐day readmission. Patients with delirium are known to be more likely to develop depressive symptoms during their hospital stay^27^ and depression has also been cited as a risk factor for postoperative delirium.^28^ If both conditions overlap, patients have a higher odds of new nursing home placement (five fold higher) and death (three fold higher) at 1 year and functional decline within 1 month compared to patients with neither condition.^29^ The odds for the aforementioned complications were further higher for the overlap syndrome compared with depression or delirium alone. Hence, the two syndromes may therefore be related, and stress, inflammatory responses, and neurotransmitter disturbances are candidates for common underlying pathways.

Delirium superimposed on dementia was associated with a lower rehospitalisation risk, contrary to expectations. However, it is possible that actively making this diagnosis (i.e., recognising that the delirium has occurred on the background of dementia) reflected or resulted in more assertive aftercare that accounted for reduced readmission. Similarly, a cognitive problem at discharge recorded on the HoNOS instrument was also associated with a lower risk of readmission, which could also be related to better care planning. Another explanation might be that delirium superimposed on dementia reflects a lower severity precipitating disorder (because of the presence of dementia as a predisposing factor).

Considering the lower readmission rates in those with alcohol/substance use problems, this might reflect a more reversible type of index delirium episode (e.g., delirium tremens), or other pathophysiological pathways and socioeconomic issues might be involved. Of relevance to our finding that patients with circulatory diseases had a higher readmission risk, Uthamalingam et al. reported that, in older patients with acute decompensated heart failure, acute delirium was associated with increased risk of 30‐day and 90‐day hospital readmissions with that disorder.^30^ That study highlighted that the increased risk was particularly high for 30‐day compared with 90‐day readmissions, and patients with delirium had an increased heart rate, increased brain natriuretic peptide, increased rales in the lung base, and a higher prevalence of impaired LV systolic function, which is in line with the idea that inflammation, stress, neurohormonal dysregulation, as well as periodic or sustained hypoxia during surgery are key factors in delirium pathogenesis.^31^, ^32^, ^33^

Our finding that polypharmacy per se was not a statistically significant risk factor was unexpected, also given that polypharmacy is a risk factor for emergency department attendance and hospital admission in people with dementia.^22^ The polypharmacy encountered in our sample of patients with delirium might be appropriate and necessary. Further, we did not distinguish the different types of delirium, and it was recently shown that medications with antimuscarinic properties are associated with a considerably higher risk of drug‐induced delirium, it is important to differentiate between different types of delirium.^34^ Receipt of an acetylcholinesterase inhibitor was associated with lower odds of 30‐day readmission, whereby this association was attenuated to a non‐significant trend after comprehensive confounder adjustment. This finding is in line with previous research showing that antidementia medications are associated with lower risk of rehospitalisation.^35^ The beneficial effects of acetylcholinesterase inhibitors on outcomes as hospitalisation, mortality and circulatory disease seen in observational studies are probably because to a combination of the cognitive and physiological effects of these medications and a bias by indication as patients started on acetylcholinesterase inhibitors are usually healthier.^36^, ^37^ This also explains the attenuation of the effect on readmission after delirium after adjustment for other factors including mental and physical health.

Strength of our study include the relatively large sample size of patients receiving routine care with an expert delirium diagnosis by general hospital psychiatric liaison services. This approach however also comes with a number of limitations. First, patients referred to mental health services might have a more severe form of delirium and thereby not representative of all patients with delirium. Second, although diagnoses of delirium were made according to ICD‐10 criteria, we cannot ascertain if structured screening or assessment tools for delirium were used. Third, we only had limited information on the nature and aetiology of the delirium, and can only report estimates for delirium in general. Fourth, we did not assess the impact of the duration of delirium and whether this is a risk factor for readmission to the hospital, and this which would be worthwhile topics for further research. Fifth, the assessment of depressed mood used the HoNOS score, which is a routine tool used in UK clinical service, but not a structured ICD‐10 interview. While the scale has not been developed specifically for people with dementia, its mental health symptom subscales have been shown to predict hospitalisation and mortality in this population.^38^, ^39^ Sixth, we excluded patients who died outside hospital within 30 days, whereby 8.5% of 2999 patients identified with a delirium admission died in hospital or the community within 30 days of being discharged. This shows that early mortality after hospital discharge is not uncommon in people admitted with delirium and, although it is beyond the scope of this study, should be explored in future research. Lastly, although the richness of our data allowed adjust for a range of demographic, functional, social, and clinical covariates, residual confounding can never be excluded in an observational study.

To conclude, we identified a number of risk factors for re‐admission following an episode of delirium, which were related to co‐morbid mental and physical health symptoms, as well as the nature and aetiology of the delirium. Screening for such risk factors and for example identifying depressive symptoms and treating them accordingly seem feasible approaches and might help to reduce the risk for readmissions, although further evaluation and research into understanding the underlying pathways is important. Almost 30% of patients with delirium diagnosed by mental healthcare liaison services were readmitted within 30 days after discharge it is evident that this topic is an important issue for health services, as well as for the large number of patients and families implicated. Targeted prevention strategies could potentially be developed and implemented prior to discharge after a delirium episode. Establishing delirium aftercare pathways might be another useful intervention to reduce the aforementioned risks.

## FUNDING INFORMATION

The data resource, Gayan Perera, Christoph Mueller and Robert Stewart are part‐funded by the National Institute for Health Research (NIHR) Biomedical Research Centre at South London and Maudsley NHS Foundation Trust and King's College London, and Robert Stewart by the National Institute for Health Research (NIHR) Applied Research Collaboration South London (NIHR ARC South London) at King's College Hospital NHS Foundation Trust, and by the DATAMIND HDR UK Mental Health Data Hub (MRC grant MR/W014386). The views expressed are those of the authors and not necessarily those of the NHS, the NIHR or the Department of Health.

## CONFLICT OF INTEREST

Robert Stewart has received recent research support in the last 36 months from Janssen, GSK and Takeda. Gayan Perera, Christoph Mueller, Lisa Leutgeb, David Haardt and Michaela‐Elena Friedrich declare no conflict of interest.

### PEER REVIEW

The peer review history for this article is available at https://publons.com/publon/10.1111/acps.13523.

## Data Availability

All relevant aggregate data are found within the paper. The data used in this work have been obtained from the Clinical Record Interactive Search (CRIS), a system that has been developed for use within the NIHR Mental Health Biomedical Research Centre (BRC) at the South London and Maudsley NHS Foundation Trust (SLaM). It provides authorised researchers with regulated access to anonymised information extracted from SLaM's electronic clinical records system. Individual‐level data are restricted in accordance to the strict patient led governance established at South London and The Maudsley NHS Foundation Trust, and by NHS Digital for the case of linked data. Data are available for researchers who meet the criteria for access to this restricted data: (1) SLaM employees or (2) those having an honorary contract or letter of access from the trust. For further details, and to obtain an honorary research contract or letter of access, contact the CRIS Administrator at cris.administrator@kcl.ac.uk.
